# Synergies of Multiple Zeitgebers Tune Entrainment

**DOI:** 10.3389/fnetp.2021.803011

**Published:** 2022-01-18

**Authors:** Saskia Grabe, Elmir Mahammadov, Marta Del Olmo, Hanspeter Herzel

**Affiliations:** ^1^ CharitéCenter for Basic Sciences, Institute for Theoretical Biology, Charité—Universitätsmedizin Berlin, Berlin, Germany; ^2^ Stem Cell Center (SCC), Institute of Epigenetics and Stem Cells, Helmholtz Zentrum München, Munich, Germany; ^3^ Department of Biology, Institute for Theoretical Biology, Humboldt-Universität zu Berlin, Berlin, Germany

**Keywords:** circadian clock, synchronisation, mathematical model, zeitgeber synergy, resonance

## Abstract

Circadian rhythms are biological rhythms with a period close to 24 h. They become entrained to the Earth’s solar day *via* different periodic cues, so-called zeitgebers. The entrainment of circadian rhythms to a single zeitgeber was investigated in many mathematical clock models of different levels of complexity, ranging from the Poincaré oscillator and the Goodwin model to biologically more detailed models of multiple transcriptional translational feedback loops. However, circadian rhythms are exposed to multiple coexisting zeitgebers in nature. Therefore, we study synergistic effects of two coexisting zeitgebers on different components of the circadian clock. We investigate the induction of *period* genes by light together with modulations of nuclear receptor activities by drugs and metabolism. Our results show that the entrainment of a circadian rhythm to two coexisting zeitgebers depends strongly on the phase difference between the two zeitgebers. Synergistic interactions of zeitgebers can strengthen diurnal rhythms to reduce detrimental effects of shift-work and jet lag. Medical treatment strategies which aim for stable circadian rhythms should consider interactions of multiple zeitgebers.

## 1 Introduction

### 1.1 Entrainment of Circadian Rhythms

The synchronisation of external rhythms (“zeitgebers”) with endogenous circadian clocks is a central topic in chronobiology (Aschoff and Pohl, 1978). Zeitgeber signals with period *T* (on Earth typically 24 h) interact with intrinsic rhythms characterised by their free running periods *τ*. Under most physiological conditions, the intrinsic period *τ* adapts to the zeitgeber period *T*, a phenomenon referred to as entrainment. In cases of entrainment, the inner rhythm adapts a stable phase relationship with respect to the Zeitgeber known as phase of entrainment *ψ*. It is this phase of entrainment that allows synchronisation of rhythmic physiological processes (Rensing and Ruoff, 2002; Dunlap et al., 2004; Yan et al., 2008) to rhythmic environments (light, temperature, nutrition).

Most experimental and theoretical studies focus on the entrainment by a single zeitgeber such as light (Pittendrigh and Daan, 1976; Wright et al., 2013). Here we study systematically synergistic effects of two zeitgebers since in many applications additional clock inputs beyond light are relevant. We analyse mathematical models of the mammalian circadian clock of intermediate complexity with at least five clock genes allowing modulations of different feedback loops.

### 1.2 Core Clock in Mammals

In most organisms the intrinsic circadian clock consists of delayed negative transcriptional translational feedback loops (Cox and Takahashi, 2019). In mammals, the transcription factors BMAL1 and CLOCK activate hundreds of target genes *via* E-boxes including *Period* and *Cryptochrome* genes (Paquet et al., 2008; Koike et al., 2012). After a delay of about 6 h the proteins of the *Per* and *Cry* genes inhibit their own transcription. Knock-out studies show that this delayed negative feedback loop is essential for the generation of activity rhythms in mammals (Van Der Horst et al., 1999; Zheng et al., 2001).

BMAL1 and CLOCK also induce transcription of the nuclear receptors ROR and Rev-Erb *via* E-boxes. ROR and Rev-erb regulate Bmal1 transcription *via* ROR-elements in the Bmal1 promoter, forming additional feedback loops (Preitner et al., 2002; Ueda et al., 2002). Knock-outs of the inhibitors Rev-Erb*α* and Rev-Erb*β* disturb rhythmicity in locomotor activity patterns and hepatic gene expression in mice (Stratmann and Schibler, 2012) pointing to the relevance of these loops. Thus, the core clock in mammals is composed of several positive and negative feedback loops.

Many zeitgebers activate the transcription of core clock genes, such as light induces *Period* transcription *via* the binding of cAMP response element-binding proteins (CREBs) at cAMP response elements (CREs) in the promoter of *Period* (Brenna et al., 2021). Other Zeitgebers are potent drugs that can control nuclear receptor levels and activity. For example, the agonist SR9009 has been shown to regulate the Rev-Erb*α* loop (Solt et al., 2012). Moreover, agonists and antagonists of Rev-Erb*α* are applied in cancer treatment. Consequently, their effects on circadian rhythms play a role in chronotherapy (Innominato et al., 2014; Dong et al., 2019).

### 1.3 Synergies of Zeitgebers

In the wild, daily rhythms of light, temperature, food availability, and ecological factors (predators, parasites, prey) serve as zeitgebers. For example, flies are entrained by phases-shifted cycles of light and temperature (Watari and Tanaka, 2010; Nikhil et al., 2014). In biomedical applications, light, meals, activities, and drugs can phase shift the endogenous clock. For instance, light and melatonin time cues can advance the human circadian clock (Wright et al., 2013) and temporal conflicts between the light-dark schedule and meal timing can result in tissue specific phase shifts, leading to desynchronised peripheral clocks (Mukherji et al., 2015; Manella et al., 2021). It was shown in mice that the combined application of light input and an CK1*δ*/*ϵ* inhibitor to Period can control circadian phases in a mathematically predictable way (Kim et al., 2013).

In our study, we examine the induction of Period expression together with modulated Rev-Erb levels, e.g., *via* light inputs and drug administration (Miller and Hirota, 2020). We simulate models of gene-regulatory networks that include the feedback loops associated to these core clock genes.

### 1.4 Data-Based Mathematical Models of Intermediate Complexity

Many previous studies of entrainment are based on amplitude-phase oscillators (Schmal et al., 2015). About 20 years ago more detailed models were derived (Forger and Peskin, 2003; Leloup and Goldbeter, 2003; Becker-Weimann et al., 2004). More recently, the important role of the Rev-Erb*α*-loop was predicted by an expanded model (Relógio et al., 2011). For our purpose, relatively simple core clock models containing the most important loops are appropriate.

Korenčič et al. (2012), Korenčič et al. (2014) developed a 6-gene model based on carefully normalized gene expression levels in mouse liver and adrenal gland. Recently, a model of seven proteins was fitted to experimental data from a mammalian cell line (Almeida et al., 2020b). Both models contain Period and Rev-Erb*α* as important feedback regulators. We study periodic modulations of these genes using adapted versions of Korenčič- and Almeida models (see Section 4).

## 2 Results

### 2.1 Proteins and Regulators Replace Explicit Delays

The Korenčič model is based on delay differential equations (DDEs). Even though DDEs require relatively few parameters they exhibit an infinite dimensional phase space (Mackey and Glass, 1977). As described in methods, we developed a unique mapping of DDE models to sets of ordinary differential equations (ODEs) to reduce the mathematical complexity.

In the classical Goodwin model, a gene *x* is translated into a protein *y*, which generates an inhibitor *z* (Ruoff and Rensing, 1996; Gonze and Ruoff, 2020). In this way a delayed negative feedback is formed. For sufficiently strong non-linearities (Griffith, 1968) this regulatory loop exhibits self-sustained oscillations. In delay-equations $\frac{dx}{dt}=f\left(x\left(t-\Delta t\right)\right)-d*x$ with explicit delays Δ*t*, limit cycles are possible for decreasing functions *f* (negative feedback) and for sufficiently long delays Δ*t* (Mackey and Glass, 1977; Korenčič et al., 2012).

In our modified Korenčič model (see Section 4 for details) we combine both approaches. The explicit delay Δ*t* is replaced by the chain of reactions from *x* to *y* to *z* (so-called ‘chain trick’ (MacDonald, 1978; Hale and Lunel, 1993; Bordyugov et al., 2013)). Biologically, *x* denotes a clock gene transcript, this clock gene transcript is translated into the clock protein *y*. The clock protein is part of the regulatory complex *z*, which regulates the transcription of *x*.

The implicit delay is governed by the degradation rates of *y* and *z*. In linear chains, analytical expressions can be derived connecting explicit delays Δ*t* with degradation rates. Thus, we can replace the delays in the Korenčič model and obtain for each gene ODEs of the form

$$\begin{array}{ccc} \frac{dx}{dt} & = & f\left(z\right)-d_{x}*x \end{array}$$ (1)

$$\begin{array}{ccc} \frac{dy}{dt} & = & x-d_{y}*y \end{array}$$ (2)

$$\begin{array}{ccc} \frac{dz}{dt} & = & y-d_{z}*z \end{array}$$ (3)

For the modelling assumption *d*_*y*_ = *d*_*z*_ = *d* we obtain just a single new parameter *d*, so the original delay parameter Δ*t* can be replaced uniquely by this new parameter *d*.

In previous publications multiple DDE models have been fitted to expression profiles of core clock genes (Korenčič et al., 2012, 2014; Pett et al., 2016). By replacing the explicit delays Δ*t* by degradation rates *d* we can derive ODE models in a straightforward way. Even without parameter tuning many of the associated ODE models exhibit oscillations (Mahammadov, 2018). We use one of these models (Supplementary Equations S1-5.1–S1-5.15), which is based on the parameter set “liver LD” (Korenčič et al., 2014), to study entrainment by two zeitgebers. The liver receives light signals from the retina *via* sympathetic innervation from the SCN, which results in the activation of *Period* expression by adrenaline/noradrenaline (Terazono et al., 2003), and *via* glucocorticoid signalling, which also alters *Period* expression (Pezük et al., 2012).

**FIGURE 1:**
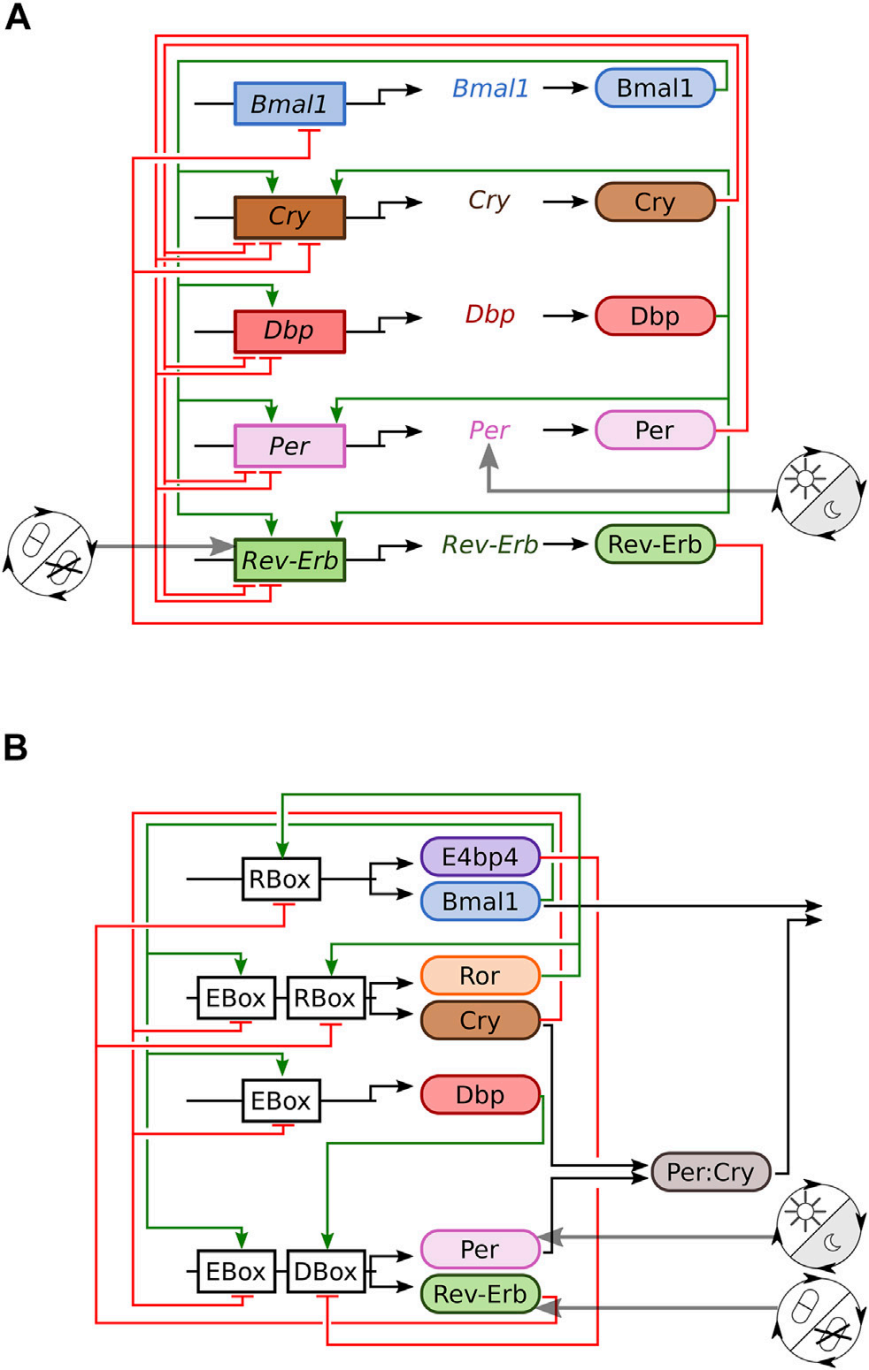
Sketches of the two investigated mammalian clock models. **(A)** Transcriptional translational feedback loops of the modified Korenčič model. **(B)** Gene-regulatory network of the Almeida model (Almeida et al., 2020b).

Figure 1A shows the resulting gene-regulatory network derived from carefully normalised gene expression profiles. It includes inhibitions (red) and activations (green) from multiple feedback loops (Pett et al., 2016). The effects of the zeitgebers are included as regulation of the *Per* gene by light and regulation of the *Rev-Erb* gene by drugs.

In order to test the generality of our results we simulate also a recently developed regulatory network of clock proteins (Almeida et al., 2020b). The network structure is displayed in Figure 1B and involves E-boxes, R-boxes and D-boxes. Zeitgebers are modelled by the modulation of PER and REV-ERB production as shown in the Supplementary Equations S1-5.16–S1-5.19.

### 2.2 Light and Drugs Entrain the Clock

Circadian rhythms become entrained to zeitgebers with periods *T* which are close to their free running periods *τ*. “Stronger” zeitgebers can entrain circadian rhythms to a larger range of periods (Abraham et al., 2010). The mammalian circadian clock has a free running period of about 24 h and without special entrainment protocols it can become entrained to zeitgeber periods within an entrainment range of about ± 1–2 h (Scheer et al., 2007; Walbeek and Gorman, 2017).

**FIGURE 2:**
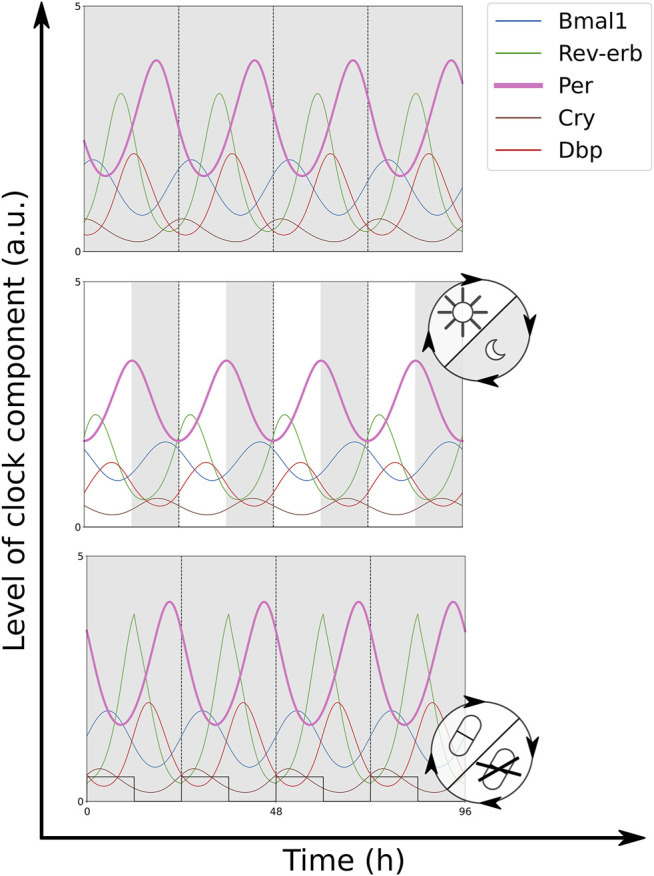
Time traces of the modified Korenčič model in constant darkness (DD) **(top)**, 12:12 light-dark cycles (LD) (middle, grey bars indicate the dark phase) and 12:12 drug—no drug cycles (bottom, drug presence visualised as square wave). Both zeitgebers result in successful entrainment to a period of 24 h. The simulation of a Rev-Erb*α* agonist (+REV) was chosen as drug. For the equations and parameter values of the simulations Supplementary Equation S1-1. All plots show *Bmal*1_*x*_, *Rev*−*erb*_*x*_, *Per*_*x*_, *Cry*_*x*_, and *DBP*_*x*_.

When each of the zeitgebers is applied solitarily, both models discussed in this paper entrain to rhythmic light inputs on Period or rhythmic drug inputs on Rev-Erb. The oscillatory output of a successfully entrained clock model oscillates with the same period as the zeitgeber rhythm (Figure 2). Matching periods are accompanied by a fixed phase relationship between the two oscillators, e.g. fixed temporal differences between zeitgeber onset and the time points when the oscillatory clock component levels cross their means. The resulting phase relationship is quantified by the phase of entrainment *ψ*.

**FIGURE 3:**
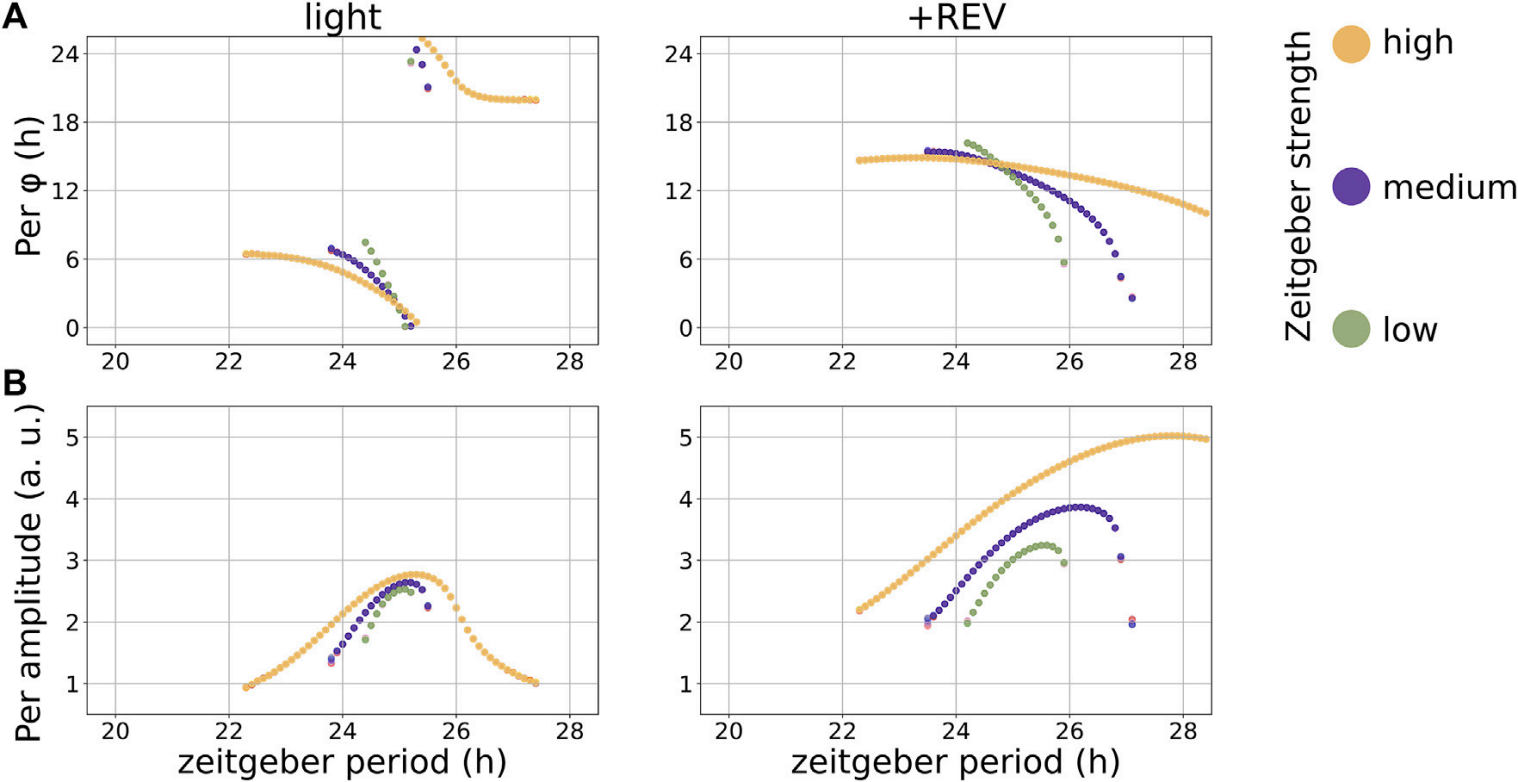
Entrainment to solitary zeitgebers in the modified Korenčič model, the zeitgeber period controls the phase of entrainment **(A)** and the entrained amplitude **(B)**. The free running period of the model is 24.8 h. Per *φ* denotes the phase of entrainment. The chosen zeitgeber strengths are *r*_*light*_ = (0.1, 0.2, 0.4) and *r*_+*REV*_ = (0.1, 0.2, 0.4).

In the modified Korenčič model, the range of entrainment to light cycles at *r*_*light*_ = 0.2 spans 1.7 h. The rhythmically applied Rev-Erb agonist, *r*_+*REV*_ = 0.2, covers a larger range of entrainment of 3.6 h (Figure 3A). A Rev-Erb antagonist of equal zeitgeber amplitude is a slightly weaker zeitgeber with a narrower range of entrainment of 2.8 h (Supplementary Figure S3-1). The period of the zeitgeber also affects the amplitude of the entrained zeitgeber rhythm, which is referred to as amplitude resonance. There is a maximum in the entrained amplitude at 25 h for light cycles and around 26 h for drug cycles as solitary zeitgeber (Figure 3B).

It is widely known that the entrainment phase depends on the zeitgeber period (Aschoff and Pohl, 1978; Schmal et al., 2015). In many cases the phase of entrainment spans a range of 180° (or 12 h) within the range of entrainment. This “12 h rule” is approximately fulfilled in both models and for all zeitgeber inputs (Figure 3 and Supplementary Figure S3-1). The upper graph of Figure 3 illustrates the large phase variations within the entrainment ranges. Consistent with oscillator theory (Granada et al., 2013) large periods lead to earlier phases. An increase in zeitgeber amplitude increases the range of entrainment and results in a weaker slope of the phases of entrainment versus zeitgeber period. Mathematically, entrainment phenomena are described *via* Arnold tongues (Schmal et al., 2015; Mosheiff et al., 2018). The phase distribution within an Arnold tongue becomes wider for higher zeitgeber strengths, resulting in the aforementioned smaller slope of the phases of entrainment at higher zeitgeber amplitudes.

In the Almeida model we observe period doubling for long driving periods at large zeitgeber amplitudes (Supplementary Figure S3-1). At these zeitgeber periods the limit cycle of the entrained circadian rhythm is folded, resulting in alternating amplitudes and alternating peak phases. Interestingly, period doubling has been also described in the autonomous Almeida model for varying degradation rates of Cry (van Soest et al., 2020).

### 2.3 The Phase Difference of Coexisting Zeitgebers Controls Entrainment

**FIGURE 4:**
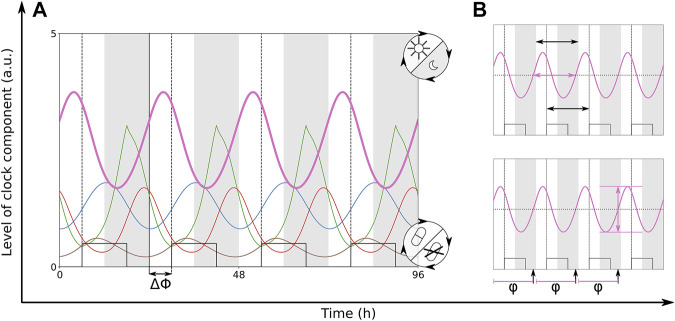
**(A)** Time traces of the modified Korenčič model, which is entrained to two coexisting zeitgebers with periods of 24 h. Dark phases are indicated *via* grey bars and drug level is shown as square wave. There is a phase difference ΔΦ = 6 h between zeitgeber onsets. **(B)** The small graphs illustrate the frequency locking at a period of 24 h (black horizontal arrows), the resulting amplitude of Per (pink vertical arrow) and the phase *φ* (black vertical arrows) which is measured when the rising Per level is crossing the mean Per level. Light is turned on within the white bars. The solid, black vertical line highlights the onset of light. Dashed, black vertical lines highlight the onset of drug presence. Colours of the clock components are given in Figure 2.

Under natural conditions (Oda and Friesen, 2011) and also in clinical environments (Innominato et al., 2014; Lachmann et al., 2021), the co-occurrence of multiple zeitgeber inputs to the circadian clock is very common. We simulate the co-occurring zeitgeber inputs of light cycles on Period and drug cycles on Rev-Erb. If both zeitgebers have the same period of 24 h their relative strength and their phase difference ΔΦ are the most essential parameters. ΔΦ is the temporal difference between the onset of light and the onset of drug presence, at ΔΦ = 0 the zeitgeber cycles are aligned. In this section, we study how the entrainment properties (period, amplitude and phase of the circadian rhythm) are controlled by coexisting zeitgebers. In Figure 4 we show the oscillations of the entrained circadian rhythm with a zeitgeber phase difference of 6 h. The common abbreviation for the entrainment phase is *ψ*. In our simulation studies, we take the temporal difference between the time point when the oscillating Per level crosses its mean and the light onset as entrainment phase termed Per *φ*.

**FIGURE 5:**
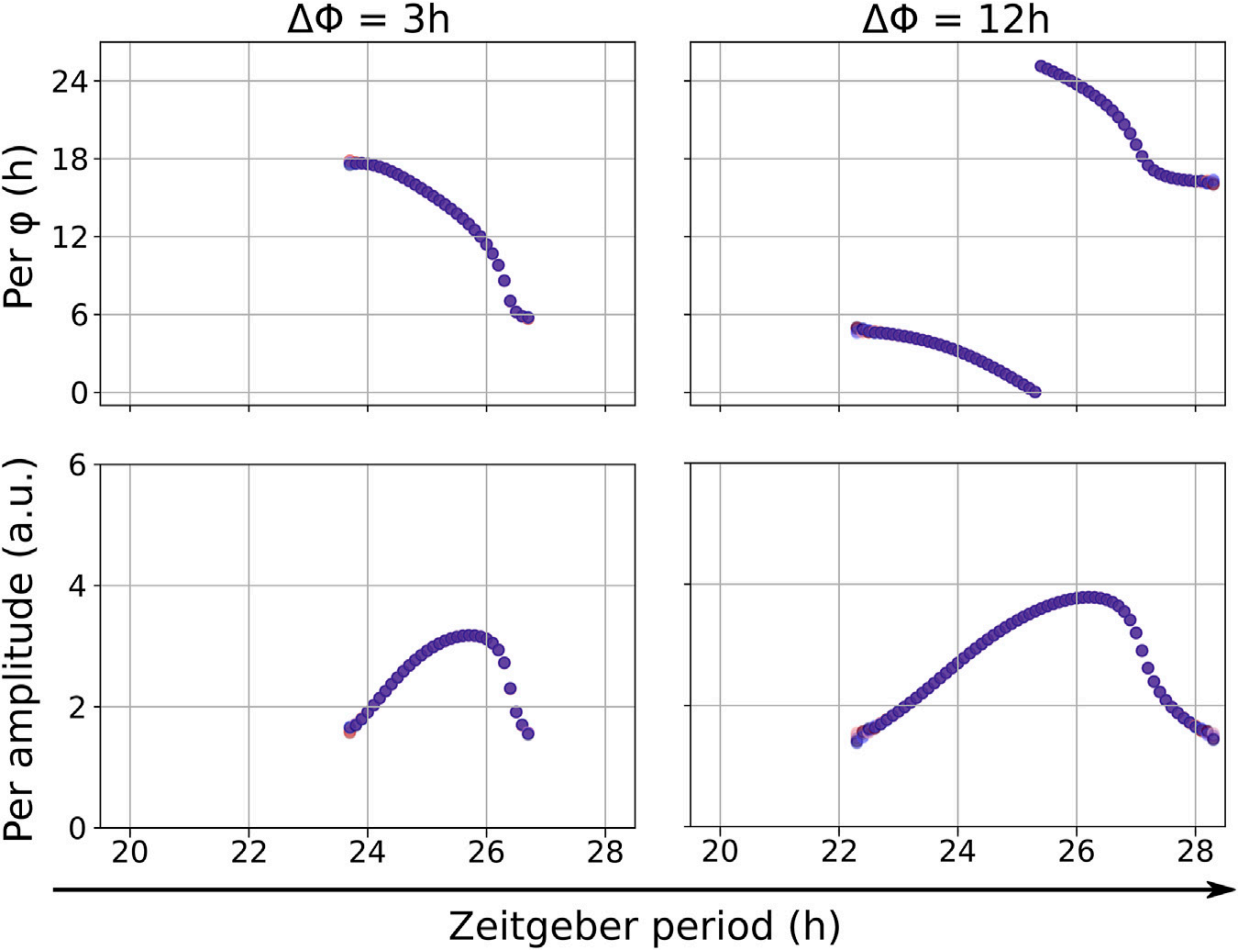
Entrainment of the modified Korenčič model to two coexisting zeitgebers (light and REV agonist) with equal zeitgeber periods. The phase differences between zeitgeber onsets ΔΦ is varied. For ΔΦ = 12 h we find a zeitgeber synergy resulting in a broad range of entrainment which is accompanied by amplitude resonance. For ΔΦ = 3 h there is a smaller range of entrainment.

When both zeitgebers exhibit no phase difference, there is successful entrainment in both models (Supplementary Figure S3-2). Typically, the range of entrainment of two co-occurring zeitgebers increases compared to the ranges of entrainment of the solitary zeitgebers. We also find that the 12 h rule applies to all investigated cases of entrainment by two coexisting zeitgebers. In the modified Korenčič model, there is a pronounced resonance of the entrained amplitude to the period of the co-occurring zeitgebers. It resembles the resonance of the circadian rhythm to the solitary zeitgeber input on Rev-Erb (compare Figures 3, 4, 5). For the Almeida model, the resonances are relatively weak and for large zeitgeber strength and long periods period-doubling occurs (Supplementary Figure S3-2).

However, the range of entrainment of two coexisting zeitgebers is governed by the phase difference between the two zeitgebers. There are synergistic phase differences between the two zeitgebers, which result in larger ranges of entrainment than for solitary zeitgebers, and antagonistic phase differences with small ranges of entrainment (Supplementary Figure S3-3). The effect of synergistic and antagonistic phase differences is also predictable from possible overlays of the phase response curves (PRCs) of the solitary zeitgebers (Supplementary Figure S3-4). For the largest zeitgeber synergy, which means almost full alignment of the two PRCs, the PRC of +REV has to be shifted towards earlier pulse onsets by about 15.5 h. Likewise, for zeitgeber antagonism, and small overlap of the PRCs, the PRC of +REV has to be shifted about 4 h towards earlier pulse onsets. Phase differences with large entrainment ranges are associated with stronger resonances (Supplementary Figure S3-3).

### 2.4 Synergies of the Two Zeitgebers

Our systematic variations of zeitgeber properties revealed that there are optimal phase relationships leading to broad entrainment ranges and large amplitudes of the entrained circadian rhythm. This agrees with synergies of light and temperature cycles in flies (Nikhil et al., 2014). Nikhil et al. experimentally observed most robust entrainment of the flies for a four-hour phase delay of the temperature cycles.

The synergistic effects of optimal phase differences in the modified Korenčič model are illustrated in Figure 5. Comparing the more optimal phase difference of ΔΦ = 12 h with the less optimal phase difference of ΔΦ = 3 h between zeitgebers of equal periods in the modified Korenčič model we find an 1.5-fold higher amplitude for ΔΦ = 12 h and a larger entrainment range (23.8–26.8 h for ΔΦ = 3 h versus 22.2 to 28.2 for ΔΦ = 12 h).

For a chosen period of 24 h for both zeitgebers, the synergistic zeitgeber phase difference also results in a shorter transient time to entrainment. We computed the transient time to entrainment according to (Granada and Herzel, 2009) (Supplementary Figure S3-7). ΔΦ = 15.5 h results in a median transient time of nine zeitgeber cycles, whereas the median transient time at ΔΦ = 4.0 h takes 20 zeitgeber cycles. This underscores the importance of the synergistic zeitgeber phase difference for fast entrainment.

Irrespectively of the zeitgeber phase difference, a resonance of zeitgeber and intrinsic clock results always in a maximum amplitude around the free running period of the modified Korenčič model. We also noticed that all resonance curves are skewed, a non-linear phenomenon called “twist” (Myung et al., 2018; Ananthasubramaniam et al., 2020; Gabriel et al., 2021).

### 2.5 Balance of Zeitgebers Governs Entrainment Phase

**FIGURE 6:**
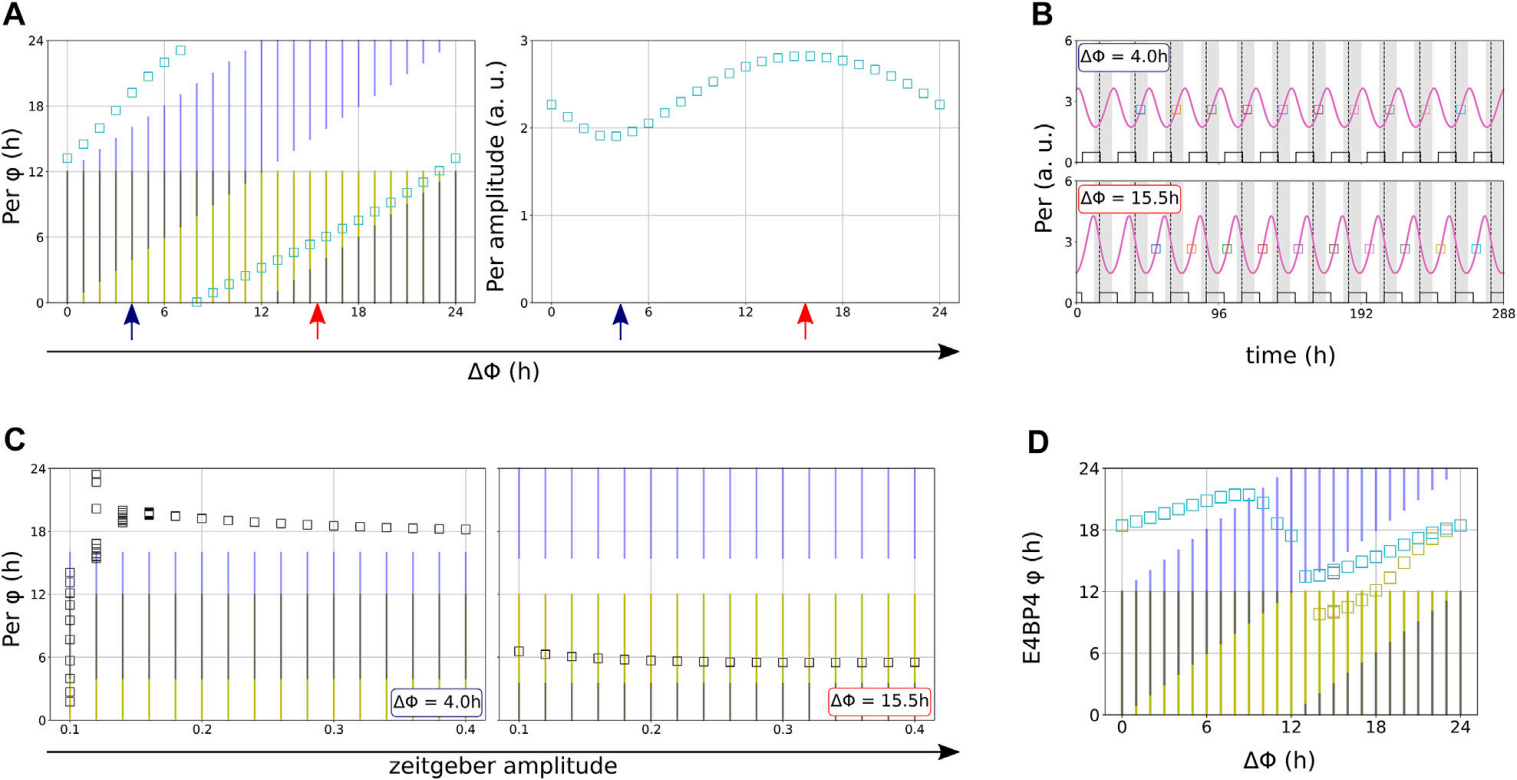
**(A)** The phase difference ΔΦ between the zeitgebers is varied in 1-h steps in the modified Korenčič model. Squares indicate the resulting entrainment phases and amplitudes. **(B)** Illustration of antagonistic (blue arrows) and synergistic effects (red arrows), also indicated by larger amplitudes. **(C)** The modified Korenčič model entrains differentially to varied amplitudes depending on the phase difference between the two zeitgebers. Note the early onset of entrainment at *r* = 0.1 for the optimal phase difference ΔΦ = 15.5 h **(D)** Entrained phases lock relative to the light onset or follow the alterations in drug onset dependent on ΔΦ in the Almeida model, some ΔΦ result in period doubling (additional yellow squares). Note that we chose to display Per for the modified Korenčič model but E4BP4 for the Almeida model because these are the clock components with the largest amplitudes. Both zeitgebers have periods of 24 h *r*_*light*_ = 0.2 and *r*_+*REV*_ = 0.2 in **(A,B)**. *r*_*light*_ = *r*_+*REV*_ are varied in **(C)**. *r*_*light*_ = 4.0, *r*_+*REV*_ = 0.04 in **(D)**. Yellow bars indicate light and blue bars indicate drug presence.

The phase of entrainment *ψ* is of central importance for the appropriate coordination of intrinsic rhythms to external zeitgebers and we have shown above that zeitgeber properties govern *ψ*. For coexisting zeitgebers with comparable strength it is not clear how the phase of entrainment is controlled. In Figure 6 we vary systematically relative zeitgeber phases ΔΦ to explore the response of the intrinsic rhythm. It turns out that entrained circadian rhythms oscillate in fixed phase relationships to the zeitgebers. We determined the phases of entrainment of the components of the clock models as temporal difference between the onset of light and the time point when the oscillations pass through their average levels (Figure 4).

The phase of entrainment of a circadian rhythm which is entrained to two coexistent zeitgebers with the same period depends on the phase difference ΔΦ between the zeitgeber onsets (Figures 6A,B) and the amplitudes *r* of the zeitgebers (Figure 6C). In the modified Korenčič model the possible phases of entrainment cover the full period of the zeitgebers (Figure 6A). Figure 6C illustrates antagonistic and synergistic interaction *via* variations of zeitgeber strength. For ΔΦ = 4 h strong zeitgebers (*r* > 0.2) are required to get entrainment. For ΔΦ = 15.5 h even *r* = 0.1 is sufficient for entrainment. The effect of zeitgeber phase differences on zeitgeber strengths is also present in the Almeida model (Supplementary Figure S3-6).

Variations of the relative phases of the two zeitgebers allows to explore the control of the entrainment phase *ϕ*. We mark the 12 h of light phase in yellow and the 12 h of drug delivery in blue. The left graph of Figure 6A reveals that the entrainment phase (green squares) follows essentially the drug delivery cycle. This is the typical outcome of simulations of the modified Korenčič model (Supplementary Figure S3-5).

In the Almeida model (Figure 6D) different phase dynamics can be found. For small ΔΦ the entrainment phase (green squares) is controlled by a mixed influence of light cycles (yellow) and drug cycles (blue). Around ΔΦ = 9 h there is a sign change of the ΔΦ dependent shifts of the phase of entrainment and for ΔΦ = 14 h period doubling occurs.

Our simulations show that the phase difference between two coexisting zeitgebers can control the phase of entrainment in semi-complex mammalian clock models. Early experimental studies with conflicting zeitgebers (Pittendrigh and Bruce, 1959; Watari and Tanaka, 2010; Oda and Friesen, 2011) revealed phase jumps comparable to our simulations in Figure 6D. Interestingly, such transitions between dominating zeitgebers have also been found in analytically solvable mathematical models (Daminelli, 2014).

**FIGURE 7:**
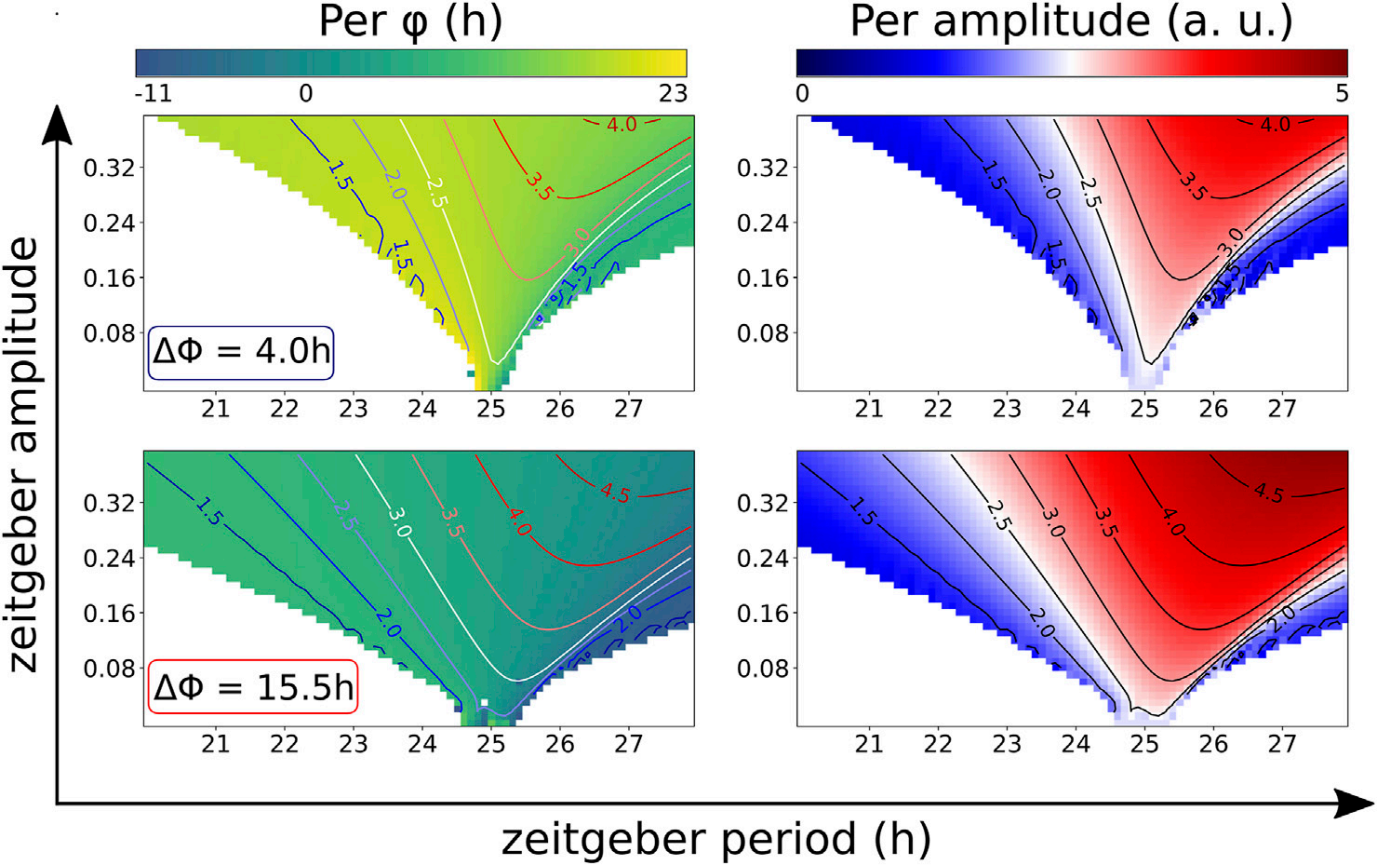
Arnold-tongues of the modified Korenčič model at synergistic (ΔΦ = 15.5 h) and antagonistic (ΔΦ = 4 h) zeitgeber differences. Both zeitgebers have the same zeitgeber periods and the same zeitgeber amplitudes *r*_*light*_ = *r*_+*REV*_. Within the region of the Arnold tongue, which is coloured according to phase of entrainment **(left plots)** or entrained amplitude **(right plots)**, there is successful entrainment with a 1:1 period ratio of zeitgeber and circadian rhythm. Isoclines highlight amplitude resonances (blue-red colour bar).

The effects of synergistic zeitgeber differences on the range of entrainment, the phases of entrainment and the entrained amplitudes are summarized in so-called Arnold-tongue diagrams (Granada et al., 2013) (Figure 7). Within the coloured area of the Arnold-tongues the circadian rhythm is successfully entrained. For the modified Korenčič model, the Arnold-tongue at the synergistic phase difference ΔΦ = 15.5 h is broader than at ΔΦ = 4 h. Compared to the Arnold-tongue at the less synergistic ΔΦ = 4 h, the Arnold-tongue at ΔΦ = 15.5 h covers more entrainable zeitgeber periods at low zeitgeber amplitudes (Figure 7, left plots). The Arnold-tongues also show the amplitude resonances, resulting in larger amplitudes of the clock component levels for long zeitgeber periods and high zeitgeber amplitudes (Figure 7, right plots).

### 2.6 Different Zeitgeber Periods Induce Large Modulations and Phase Jumps

Natural zeitgebers such as light and temperature have typically the same period of 24 h. Drug delivery, however, could deviate from a 24 h schedule. Bernard et al. (2010) investigated chronomodulated drugging of cell cycle models which are coupled to circadian clocks. They suggest a treatment interval which exceeds the period of the circadian rhythm to reduce circadian side effects of drug application at adverse phases. Specifically, a drug delivery with a period of 28.8 h, which is considered circadian-independent, was particularly efficient. Motivated by the studies by Bernard et al., we simulate light with a period of 24 h together with Rev-Erb agonist treatment cycling with 28.8 h.

**FIGURE 8:**
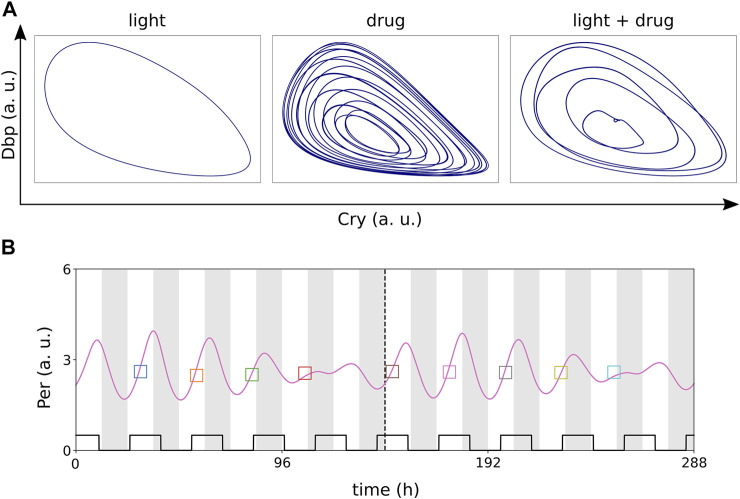
**(A)** Entrainment of the modified Korenčič model to light-dark cycles as zeitgeber with a period of 24 h or drug cycles as zeitgeber with a period of 28.8 h. Entrainment to the light-dark cycles is successful and results in a limit cycle **(left)**, but entrainment to the drug cycles results in toroidal dynamics (middle) instead. Coexisting 24 h light-dark cycles and 28.8 h drug cycles result in successful 5:6 entrainment of the circadian rhythm with “folded” limit cycles **(right)**. **(B)** Example time trace of 5:6 entrainment. Zeitgeber amplitudes in all panels: *r*_*light*_ = 0.2 and *r*_+*REV*_ = 0.2.

The suggested (Bernard et al., 2010) 5:6 ratio between the periods of the light cycle (24 h) and the drug application (28.8 h) results in 5:6 entrainment of the circadian rhythm with visible amplitude and phase modulations (Figure 8).

Figure 8A shows that light-dark cycles with *T* = 24 h can entrain the clock whereas a drug treatment with *T* = 28.8 h leads to toroidal oscillations. Applying both zeitgebers simultaneously leads to a “folded limit cycle” with 5:6 frequency locking. Phases of this folded limit cycle vary within the range of 0–12 h and amplitudes vary dramatically from day to day, exceeding even the resonance effects discussed above (Figure 8B).

## 3 Discussion

In natural and clinical environments, there are usually multiple zeitgeber rhythms, such as light (Comas et al., 2006; Lachmann et al., 2021), food availability (Woller et al., 2016; Manella et al., 2021) or drug schedules (Bernard et al., 2010; Innominato et al., 2014; Battaglin et al., 2021), acting simultaneously on the circadian clock. Additionally, on the single cell level intercellular coupling (Tokuda et al., 2018) and also cell intrinsic rhythms can interact rhythmically with components of the circadian clock. This is evident for the cell cycle (Feillet et al., 2014; Droin et al., 2019; Almeida et al., 2020a; Battaglin et al., 2021), metabolic (Woller et al., 2016) and redox rhythms (Del Olmo, 2021). Thus, with this multitude of zeitgebers that can act on the organismic and molecular clock network, it is an important goal to understand the optimal timing relationships between the different Zeitgebers. This goal becomes specially relevant in patients with disturbed clocks [including ICU patients, septic shock patients and cancer patients (Broadberry et al., 2018; Lachmann et al., 2021)], since optimal timing schedules might improve their clock function and overall their well-being. Experimentally, robust circadian rhythms *via* scheduled feeding improved the prognosis of mice with pancreatic adenocarcinoma (Li et al., 2010). It is also proposed to include specific entrainment protocols for improved circadian synchronisation into cancer therapy (Innominato et al., 2014) to maximise drug efficacy and minimise side effects. Comparable circadian schedules are also applied to facilitate entrainment after jet lag (Diekman and Bose, 2018) and to reduce adverse effects of shift work (Boivin and James, 2002). An alternative to the design of full entrainment schedules are short drug pulses to adaptively shift circadian phases at the subsequent day. This modelling approach was used to study the relative strength of the negative and the positive feedback loop of the circadian clock (Brown and Doyle, 2020) and to investigate interindividual variabilities in response to circadian drugs (Kim et al., 2013).

In this study we mathematically investigated the effect of two coexisting zeitgebers, light-dark cycles and rhythmic drug application on Rev-Erb*α*, in two models of the mammalian circadian clock, which are based on murine gene expression (Korenčič et al., 2012; Almeida et al., 2020b). We found that both zeitgebers can solitarily entrain the circadian clock and that they can also entrain the circadian clock when applied simultaneously with equal periods of both zeitgebers. There is a strong effect of the phase difference between the two zeitgeber rhythms on the entrainment. Depending on the phase difference between the two zeitgebers the phase of entrainment of the circadian rhythm is shifted together with a shift in drug onset or keeps its position relative to the light onset. Moreover, there are synergistic zeitgeber differences which result in larger entrainment ranges and amplitude resonances of the circadian rhythms. Also, the transient times are shorter at synergistic phase differences (Supplementary Figure S3-7). If the two zeitgebers do not share the same period, the circadian rhythm can also become entrained to period ratios which deviate from 1:1. These period ratios are accompanied by amplitude and phase modulations.

Our modelling results therefore predict that controlling the phase relationship between Rev-Erb modulation and light input might represent an approach by which disturbed rhythms with low circadian amplitude, e.g., in septic shock or cancer patients, could be strengthened. Nevertheless, it is important to remark that there is not a gold standard amplitude mesor yet, and thus it is not easy to define what represents a good measure of circadian amplitude. The larger range of entrainment observed at the “optimal” phase relationship between the zeitgebers points to more robust clocks, that can adapt to different, and more extreme, zeitgeber periods. In these lines, light and Rev treatment, if applied at the correct phase, might be able to entrain more extreme chronotypes such as familial advanced sleep phase syndrome (FASPS) patients or very night owls. Lastly, our simulations predict that transient perturbations of entrainment, such as jet lag, should decay faster for such “optimal” phase differences (Supplementary Figure S3-7).

At the moment experimental investigation of circadian rhythms in complex clinical environments with circadian treatment schedules is still sparse. Mathematical modelling can help to find optimal timing and strength of zeitgebers based on the dynamics of circadian rhythms within complex environments.

## 4 Methods

### 4.1 Modeling Gene Regulatory Networks

The mammalian circadian clock consists of multiple transcriptional feedback loops which are cross-regulated *via* the binding of transcription factor protein complexes at DNA response elements (E-boxes, R-boxes, and D-boxes).

Korenčič et al. (2012), Korenčič et al. (2014) developed a five DDE model of the murine circadian clock, which includes the five core clock genes Bmal1, Rev-Erb*α*, Per, Cry and Dbp. The effect of Ror is included into the dynamics of Rev-Erb*α*, because they both regulate their targets *via* R-boxes. Likewise, the effect of E4bp4 is included into the dynamics of Dbp as they both act on D-boxes.

We turned this five DDE model into a 15 ODE model (Figure 1A, Supplementary Equations S5.1–S5.15) and compare it to the eight ODE model by Almeida et al. (Figure 1B, S1 equations 5.16 to 5.19), which was rescaled to a free running period of 24.8 h (van Soest et al., 2020).

Both models are based on core clock motives of transcriptional translational feedback loops, but there are regulatory differences between the two models. Per and Cry both act as E-box repressors (Panda et al., 2002) and Cry is activated by the D-box activator Dbp (Ukai-Tadenuma et al., 2011) in Korencic et al., but Per does not act as E-box repressor and Cry is not regulated *via* D-boxes in the Almeida model (Almeida et al., 2020b).

### 4.2 Derivation of the Modified Korenčič Model

In linear reaction chains the implicit delay can be calculated analytically (Korenčič et al., 2014). This is illustrated in the Supplementary Figure S2 showing the dependency of the differences of peak phases Δmax on the half time *t*_1/2_. In delay differential equations the delayed version of a gene *x* inhibits or activates target genes.

We introduce the auxiliary variables *y* and *z* instead, representing the associated proteins and regulatory complexes. The delay of *y* and *z* with respect to the driving gene *x* is governed by their degradation rates *d*_*y*_ and *d*_*z*_. Assuming for simplicity *d*_*y*_ = *d*_*z*_ = *d*, we can replace the explicit delay Δ*t* uniquely by the degradation rate *d*. In this way, we can systematically derive 15-ODE models from the 5-DDE models as introduced in Korencic et al. (2014), Pett et al. (2016). To our surprise many of these associated ODE models display oscillations even without additional parameter tuning.

In this study we selected the experimentally based mouse liver LD parameter set by Korenčič et al. for modelling and computed the degradation rates *d*. The replacement of explicit delays by auxiliary variables *y* and *z* resulted in damped oscillations. We adjusted our degradation rates (*d*_*Bmal*_, *d*_*Rev*−*erb*_, *d*_*Per*_, *d*_*Cry*_, *d*_*Dbp*_) to obtain undamped oscillations with a free running period of 24.95 h. The resulting equations and parameters are given in Supplementary Presentation S1.

We chose light-dark cycles as zeitgeber input to Per and a rhythmically applied small molecule drug as zeitgeber input to Rev-Erb (Miller and Hirota, 2020). Both zeitgebers are modelled as square waves with amplitude *r*. The zeitgeber amplitudes are chosen to reproduce the known entrainment range of ± 1–2 h of the mammalian circadian system to a solitary zeitgeber.

All simulations were run for 80 zeitgeber cycles. Periods, phases, and amplitudes were determined across the last 10 zeitgeber cycles *via* Lombscargle periodograms (astropy lombscargle) and peak picking (own code).

## Data Availability

The original contributions presented in the study are included in the article/Supplementary Material, further inquiries can be directed to the corresponding author.
