# Supplementary material for: Synergies of Multiple Zeitgebers Tune Entrainment
Source: Front Netw Physiol. 2022 Jan 18;1:803011. doi: 10.3389/fnetp.2021.803011 (PMC10013031; doi:10.3389/fnetp.2021.803011)
Supplement: Supplementary file 1 [file Presentation1.PDF]

Supplementary Material 1

1 EQUATIONS

1.1 Modified Korenčič model

$$\frac{dBmal_x}{dt} = \left(\frac{1}{\frac{Reverb_z}{inh_{21}}+1}\right)^2 - deg_1 * Bmal_x \tag{S1}$$

$$\frac{dBmal_y}{dt} = Bmal_x - d_{Bmal} * Bmal_y \tag{S2}$$

$$\frac{dBmal_z}{dt} = Bmal_y - d_{Bmal} * Bmal_z \tag{S3}$$

$$\frac{dReverb_x}{dt} = \left(\frac{\frac{actn_{12}*Bmal_z}{act_{12}}+1}{\frac{Bmal_z}{act_{12}}+1}\right)^3 \left(\frac{1}{\frac{Per_z}{inh_{32}}+1}\right)^3 \left(\frac{\frac{actn_{52}*Dbp_z}{act_{52}}+1}{\frac{Dbp_z}{act_{52}}+1}\right)\left(\frac{1}{\frac{Cry_z}{inh_{42}}+1}\right)^3 - deg_2 * Reverb_x + Z(t)_{REV} * Reverb_x \tag{S4}$$

$$\frac{dReverb_y}{dt} = Reverb_x - d_{Reverb} * Reverb_y \tag{S5}$$

$$\frac{dReverb_z}{dt} = Reverb_y - d_{Reverb} * Reverb_z \tag{S6}$$

$$\frac{dPer_x}{dt} = \left(\frac{\frac{actn_{13}*Bmal_z}{act_{13}}+1}{\frac{Bmal_z}{act_{13}}+1}\right)^2 \left(\frac{1}{\frac{Per_z}{inh_{33}}+1}\right)^2 \left(\frac{\frac{actn_{53}*Dbp_z}{act_{53}}+1}{\frac{Dbp_z}{act_{53}}+1}\right)\left(\frac{1}{\frac{Cry_z}{inh_{43}}+1}\right)^2 - deg_3 * Per_x \tag{S7}$$

$$\frac{dPer_y}{dt} = Per_x - d_{Per} * Per_y + Z(t)_{light} \tag{S8}$$

$$\frac{dPer_z}{dt} = Per_y - d_{Per} * Per_z \tag{S9}$$

$$\frac{dCry_x}{dt} = \left(\frac{1}{\frac{Reverb_z}{inh_{24}}+1}\right)^2 \left(\frac{\frac{actn_{14}*Bmal_z}{act_{14}}+1}{\frac{Bmal_z}{act_{14}}+1}\right)^2 \left(\frac{1}{\frac{Per_z}{inh_{34}}+1}\right)^2 \left(\frac{\frac{actn_{54}*Dbp_z}{act_{54}+1}}{\frac{Dbp_z}{act_{54}}+1}\right)\left(\frac{1}{\frac{Cry_z}{inh_{44}+1}}\right)^2 - deg_4 * Cry_x \tag{S10}$$

$$\frac{dCry_y}{dt} = Cry_x - d_{Cry} * Cry_y \tag{S11}$$

$$\frac{dCry_z}{dt} = Cry_y - d_{Cry} * Cry_z \tag{S12}$$

$$\frac{dDbp_x}{dt} = \left(\frac{\frac{actn_{15}*Bmal_z}{act_{15}}+1}{\frac{Bmal_z}{act_{15}}+1}\right)^3 * \left(\frac{1}{\frac{Per_z}{inh_{35}}+1)}\right)^3 * \left(\frac{1}{\frac{Cry_z}{inh_{45}}+1}\right)^3 - deg_5 * Dbp_x \tag{S13}$$

$$\frac{dDbp_y}{dt} = Dbp_x - d_{Dbp} * Dbp_y \tag{S14}$$

$$\frac{dDbp_z}{dt} = Dbp_y - d_{Dbp} * Dbp_z \tag{S15}$$

1.2 Modified Korenčič model default parameters

$inh_{21} = 5.85, deg_1 = 0.35, d_{Bmal} = 0.65, actn_{12} = 4.34, act_{12} = 1.30, inh_{32} = 1.81, actn_{52} = 2.55, act_{52} = 0.15, inh_{42} = 103.03, deg_2 = 0.82, d_{Rev-erb} = 0.66, actn_{13} = 3.58, act_{13} = 5.20, inh_{33} = 64.66, actn_{53} = 12.74, act_{53} = 0.09, inh_{43} = 0.32, deg_3 = 0.43, d_{Per} = 0.63, inh_{24} = 1.07, actn_{14} = 1.44, act_{14} = 0.05, inh_{34} = 8.57, actn_{54} = 28.71, act_{54} = 0.72, inh_{44} = 0.68, deg_4 = 0.27, d_{Cry} = 0.49, actn_{15} = 13.65, act_{15} = 0.01, inh_{35} = 0.92, inh_{45} = 1.49, deg_5 = 0.68, d_{Dbp} = 5.02, r_{light} = 0.2, r_{-REV} = -0.2, r_{+REV} = 0.2$

1.3 Almeida model (Almeida et al., 2020)

$$\begin{aligned} EBOX &= v_e * \frac{BMAL1}{BMAL1 + k_e + k_{er} * BMAL1 * CRY} \\ RRE &= v_r * \frac{ROR}{ROR + k_r} * \frac{k_{rr}^2}{k_{rr}^2 + REV^2} \\ DBOX &= v_d * \frac{DBP}{DBP + k_d} * \frac{k_{dr}}{k_{dr} + E4BP4} \end{aligned} \tag{S16}$$

$$\begin{aligned} \frac{dBMAL1}{dt} &= RRE - \gamma_{BP} * BMAL1 * PER\_CRY \\ \frac{dROR}{dt} &= EBOX + RRE - \gamma_{ROR} * ROR \\ \frac{dREV}{dt} &= 2 * EBOX + DBOX - \gamma_{REV} * REV + Z(t)_{REV} * REV \\ \frac{dDBP}{dt} &= EBOX - \gamma_{DB} * DBP \\ \frac{dE4BP4}{dt} &= 2 * RRE - \gamma_{E4} * E4BP4 \\ \frac{dCRY}{dt} &= EBOX + 2 * RRE - \gamma_{PC} * PER * CRY + \gamma_{CP} * PER\_CRY - \gamma_c * CRY \\ \frac{dPER}{dt} &= EBOX + DBOX - \gamma_{PC} * PER * CRY + \gamma_{CP} * PER\_CRY - \gamma_P * PER + Z(t)_{light} \\ \frac{dPER\_CRY}{dt} &= \gamma_{PC} * PER * CRY - \gamma_{CP} * PER\_CRY - \gamma_{BP} * BMAL1 * PER\_CRY \end{aligned} \tag{S17}$$

1.4 Almeida model default parameters

$v_r = 44.4, k_r = 3.54, k_{rr} = 80.1, v_e = 30.3, k_e = 214., k_{er} = 1.24, v_d = 202., k_d = 5.32,$
$k_{dr} = 94.7, \gamma_{ROR} = 2.55, \gamma_{REV} = 0.4, \gamma_P = 0.844, \gamma_c = 2.34, \gamma_{DB} = 0.156,$
$\gamma_{E4} = 0.295, \gamma_{PC} = 0.191, \gamma_{CP} = 0.141, \gamma_{BP} = 2.58,$
$r_{light} = 4.0, r_{-REV} = -0.04, r_{+REV} = 0.04$

1.5 Zeitgebers

$$Z(t)_{light} = \begin{cases} 0, & \text{if } (t + T/2) mod(T) \geq T/2 \\ r_{light}, & \text{else} \end{cases} \tag{S18}$$

$$Z(t)_{REV} = \begin{cases} 0, & \text{if } (t - \Phi + T/2) mod(T) \geq T/2 \\ r_{REV}, & \text{else} \end{cases} \tag{S19}$$

REFERENCES

Almeida, S., Chaves, M., and Delaunay, F. (2020). Transcription-based circadian mechanism controls the duration of molecular clock states in response to signaling inputs. *Journal of Theoretical Biology*, 484:110015.
