# Supplementary material for: Synergies of Multiple Zeitgebers Tune Entrainment
Source: Front Netw Physiol. 2022 Jan 18;1:803011. doi: 10.3389/fnetp.2021.803011 (PMC10013031; doi:10.3389/fnetp.2021.803011)
Supplement: Supplementary file 2 [file Presentation2.pdf]

Supplementary Material 2

1 TURNING DDES INTO ODES

According to Korenčič et al. the peak phase differences of the oscillatory solution of $\frac{dx}{dt} = 1+cos(0.26h^{-1} * t) - d * x(t)$ depends on the half-time $t_{1/2} = \frac{log(2)}{d}$ (Korenčič et al., 2012). Thus, the maximum of a periodically driven gene is shifted by Δmax as a function of its half-life (compare Figure S2A).
Here we compare a self-oscillating model with explicit delay Δt to an adjusted Goodwin model.

DDE:

$$\frac{dX}{dt} = (\frac{1.}{c + X(t - \Delta t)})^{3} - d * X(t) \tag{S1}$$

ODE system :

$$\frac{dx}{dt} = (\frac{1.}{c + z(t)})^{10} - d * x(t) \tag{S2}$$

$$\frac{dy}{dt} = x - D * y(t) \tag{S3}$$

$$\frac{dz}{dt} = y - D * z(t) \tag{S4}$$

Figure S2B shows the associated rhythms. The delayed $X(t - \Delta t)$ can be replaced with $z(t)$ for an appropriate degradation rate D.
In this illustrative example the Hill coefficient of $\frac{dx}{dt}$ was increased from three to 10 to achieve the necessary nonlinearity for oscillations. Note that in the complete modified Korenčič model the Hill coefficients remain unchanged. The interactions between the clock components (Korenčič et al., 2012) were sufficiently nonlinear for oscillations without changes for the transformation from DDEs to ODEs.

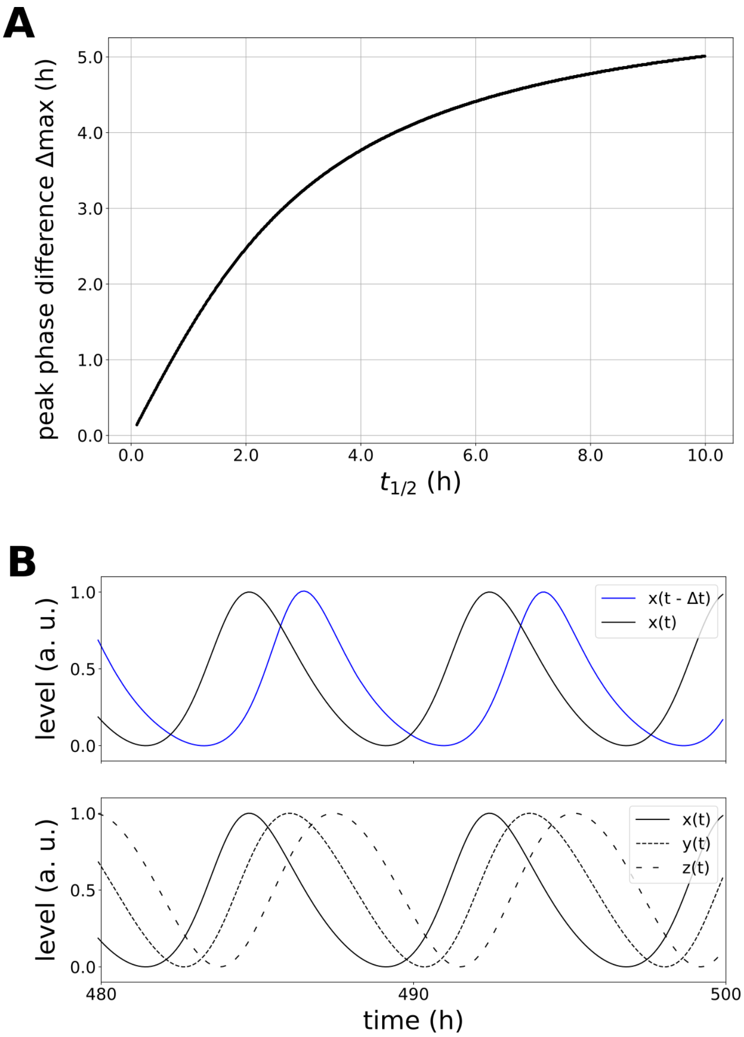

Figure S2: **(A)** Dependency of the peak phase difference of the oscillatory solution of $\frac{dx}{dt} = 1 + cos(0.26h^{-1} * t) - d * x(t)$ on the half-time $t_{1/2}$. **(B)** Time traces of the solution of the DDE $X(t - \Delta t)$ (blue, Equation S1) and the ODE system $x(t), y(t), z(t)$ (black, Equation S2-S4). All curves are normalised for display. Parameters: c=0.2, d = 0.5, the delay Δt = 2.4h was turned into D = 0.5h^{-1} according to the dependency plotted in A.

REFERENCES

Korenčič, A., Bordyugov, G., Košir, R., Rozman, D., Goličnik, M., and Herzel, H. (2012). The interplay of cis-regulatory elements rules circadian rhythms in mouse liver. *PLOS ONE*, 7(11):1–13.
