# Supplementary material for: Synergies of Multiple Zeitgebers Tune Entrainment
Source: Front Netw Physiol. 2022 Jan 18;1:803011. doi: 10.3389/fnetp.2021.803011 (PMC10013031; doi:10.3389/fnetp.2021.803011)
Supplement: Supplementary file 3 [file Presentation3.PDF]

Supplementary Material 3

1 ENTRAINMENT CHARACTERISTICS FOR ADDITIONAL MODEL VERSIONS

1.1 Ranges of entrainment to solitary zeitgebers

The modified Korenčič model and the Almeida model entrain to light cycles and drug cycles (Rev-Erbα agonist or antagonist, labeled ±REV) as solitary zeitgebers. Larger zeitgeber strengths result in larger ranges of entrainment. There are period doubling 'bubbles' for specific zeitgeber periods in the Almeida model.

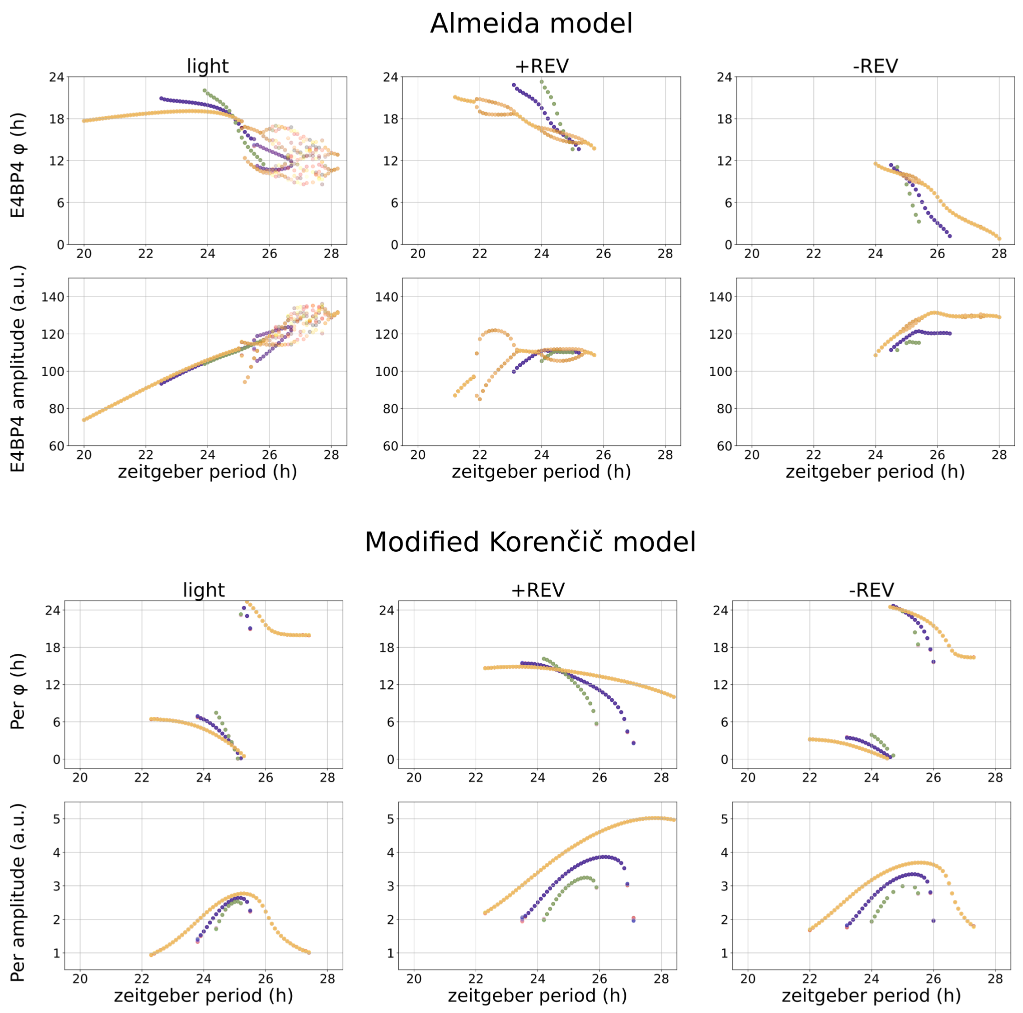

Figure S3-1: Ranges of entrainment to solitary zeitgebers. An increase in zeitgeber strength via r increases the range of entrainment. Green: $0.5 * r$, purple $1.0 * r$, orange: $2.0 * r$, the model specific and zeitgeber specific values of r are given in S1-1.

1.2 Entrainment phases and amplitudes for coexisting zeitgebers

Figure S3-2 illustrates that phase differences $\Delta\Phi$ between zeitgebers affect entrainment ranges and amplitudes. Synergies occur for $\Delta\Phi$ = 0h and +REV or $\Delta\Phi$ = 6h, 12h and -REV in the Almeida model. Note also the widespread occurrences of period doubling. For $\Delta\Phi$ = 12h and +REV or $\Delta\Phi$ = 0h, 3h, 6h and -REV we find large entrainment regions and amplitudes in the modified Korenčič model. A detailed dependence of the span of entrainable periods on $\Delta\Phi$ in the modified Korenčič model is shown in S3-3.

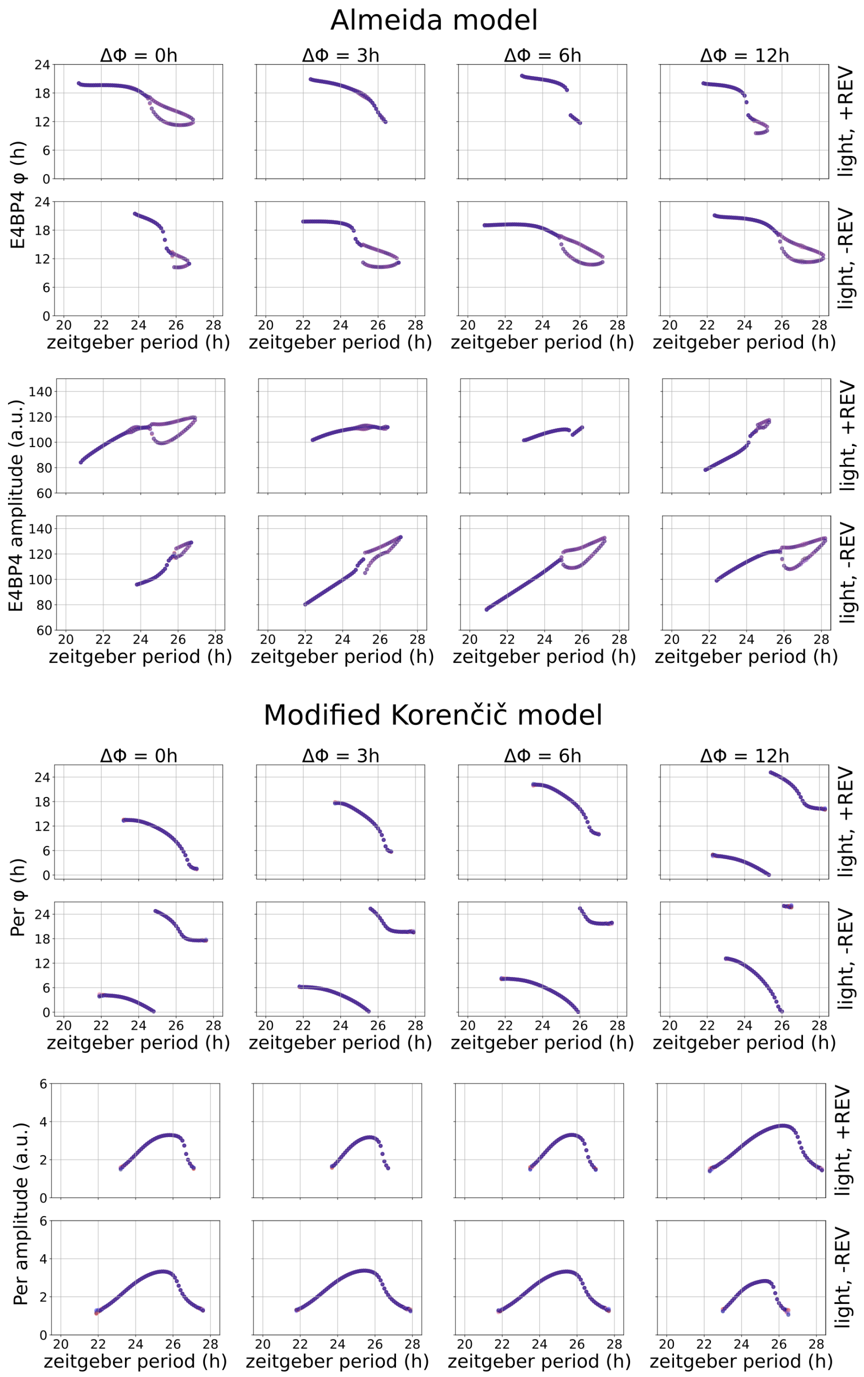

Figure S3-2: Entrainment characteristics of coexisting zeitgebers with equal periods.

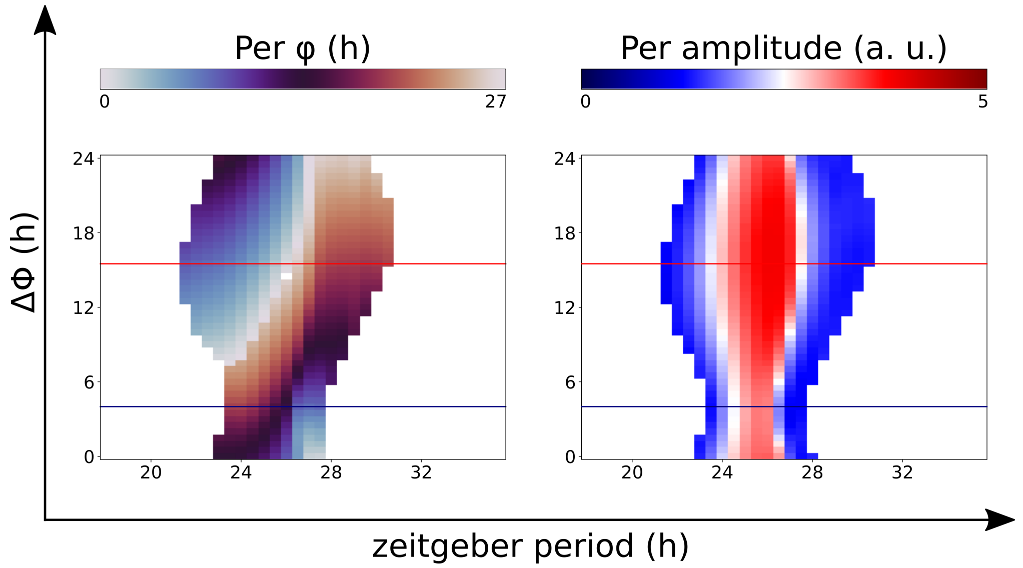

Figure S3-3: Dependence of the ranges of entrainment on zeitgeber period and $\Delta\Phi$ of two coexisting zeitgebers with equal periods in the modified Korenčič model. The phase of entrainment φ depends on $\Delta\Phi$ and the zeitgeber period (left plot). There are amplitude resonances for all $\Delta\Phi$ (right plot). $\Delta\Phi$ = 4.0h (blue) and $\Delta\Phi$ = 15.5h (red) are highlighted.

1.3 Phase response curves

To obtain a phase response curve, the free running circadian rhythm is perturbed by a single zeitgeber pulse. This results in a phase shift, when the perturbed circadian rhythm is compared to the unperturbed circadian rhythm. The difference between the maximum in phase advance (positive phase shift) and the minimum in phase delay (negative phase shift) in a phase response curve is related to the range of entrainment of its zeitgeber. Onsets of zeitgeber pulses are denoted in internal time, so they are measured relative to the free running period.

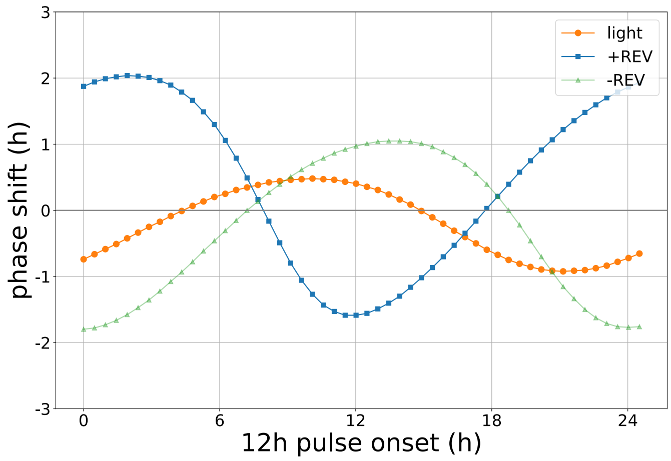

Figure S3-4: Phase response curves of the zeitgeber signals in the modified Korenčič model. The relative positions of the onsets of the 12-hour pulses within the free running period are rescaled to an external period of 24 hours.

1.4 Phases of entrainment and amplitude resonances depend on $\Delta\Phi$

The phase of entrainment (middle panels in Figure S3-5) and the entrained amplitude (right panels in Figure S3-5) depend on the zeitgeber phase difference $\Delta\Phi$ between co-occurring light cycles (yellow) and drug cycles (blue). In the Almeida model, a change in $\Delta\Phi$ can also result in period doubling (two squares) or loss of entrainment (multiple squares). Some time traces of selected $\Delta\Phi$ (1:1 entrainment, period doubling and loss of entrainment for the Almeida model and minimum versus maximum in amplitude resonance for the modified Korenčič model) are shown at the left.

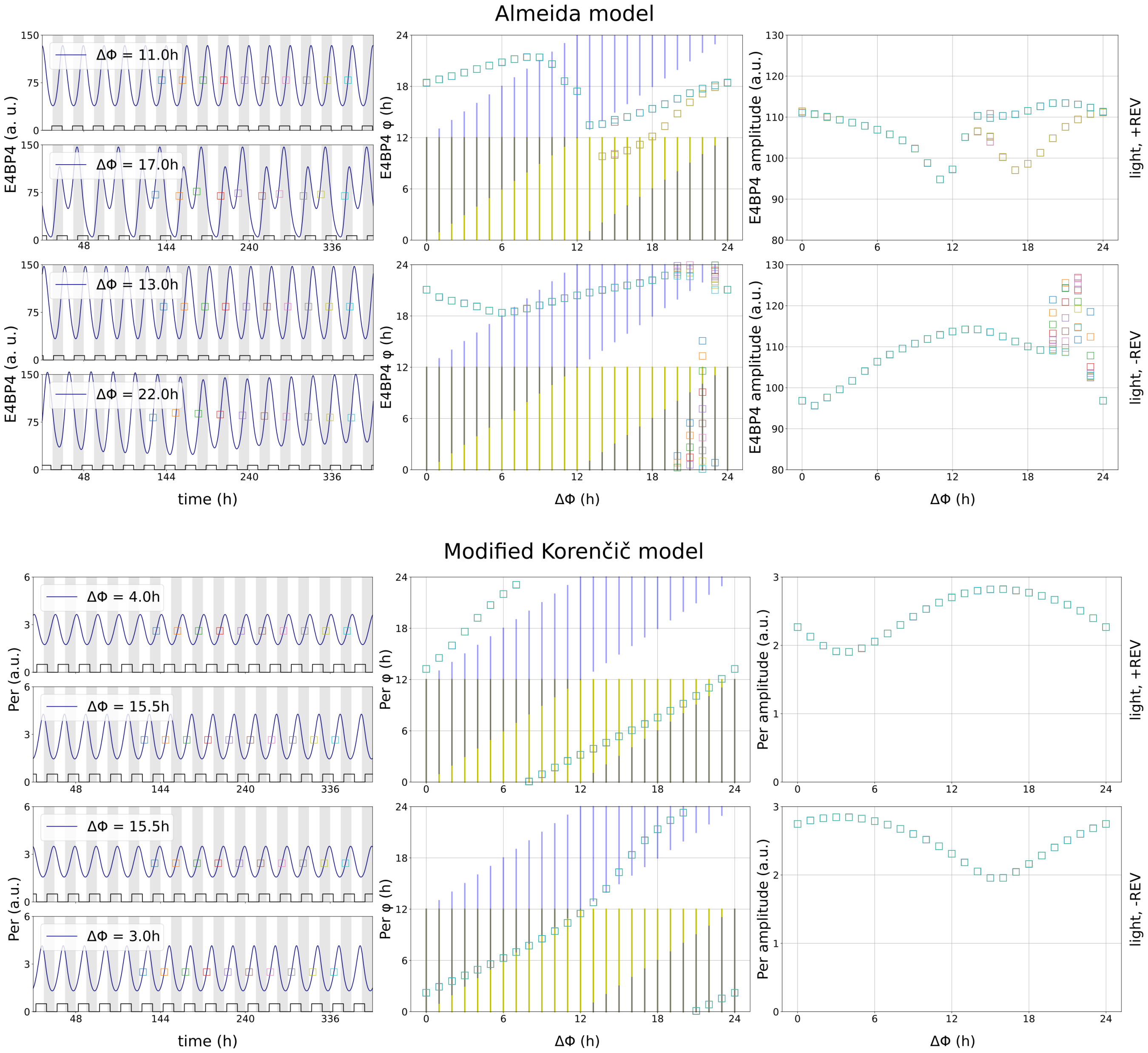

Figure S3-5: Phases of entrainment and amplitude resonances depend on the phase difference between light onset and drug presence $\Delta\Phi$. Light-dark cycles (yellow = light present) and rhythmic drug application (blue = drug present) have the same period of 24h. Rev-Erb is either activated (+REV, top plots) or inhibited (-REV, bottom plots).

1.5 Synergistic $\Delta\Phi$ facilitate entrainment

Synergistic phase differences between zeitgebers facilitate entrainment. For $\Delta\Phi$ = 17h (+REV) and $\Delta\Phi$ = 13h (-REV) in the Almeida model and $\Delta\Phi$ = 15.5h (+REV) and $\Delta\Phi$ = 4h (-REV) in the modified Korenčič model there is entrainment for reduced zeitgeber amplitudes (single squares opposed to multiple squares). The occurrence of period doubling (two squares) in the Almeida model is also dependent on the zeitgeber amplitude.

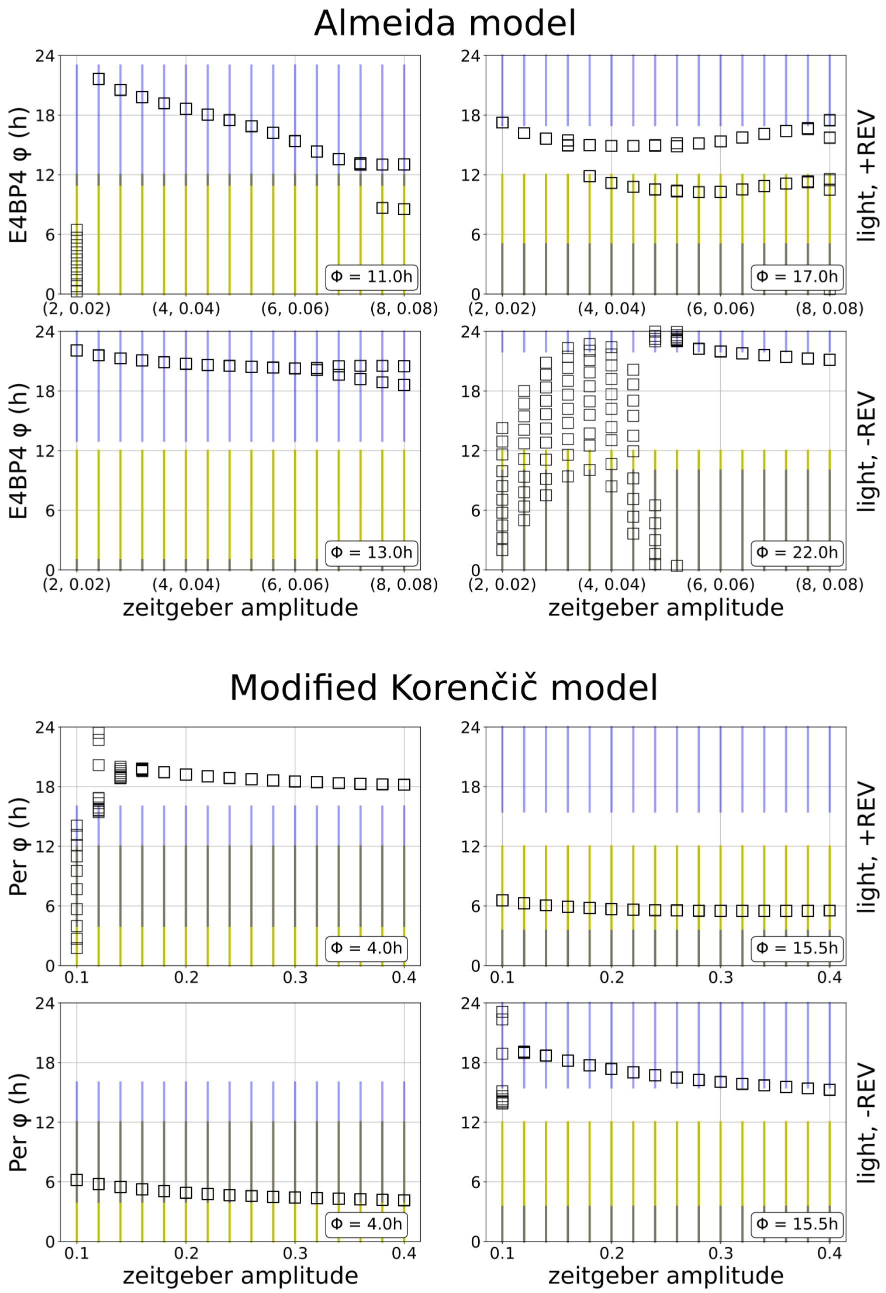

Figure S3-6: Synergistic $\Delta\Phi$ facilitate entrainment. Both zeitgebers have the same period of 24h. The zeitgeber amplitude is varied for selected synergistic and antagonistic zeitgeber phase differences $\Delta\Phi$ (see also time traces in S3-5). For the Almeida model, tuples indicate zeitgeber amplitudes of (light, REV).

1.6 Transient time to entrainment

The transient time to stable entrainment was determined according to (Granada and Herzel, 2009): the phase of entrainment is tracked across zeitgeber cycles until the mean of the phases of entrainment of eight consecutive zeitgeber cycles is below 1/12 h. In Figure S3-7 the transient time for the synergistic zeitgeber phase difference $\Delta\Phi = 15.5$h is compared to $\Delta\Phi = 4.0$h in the modified Korenčič model.

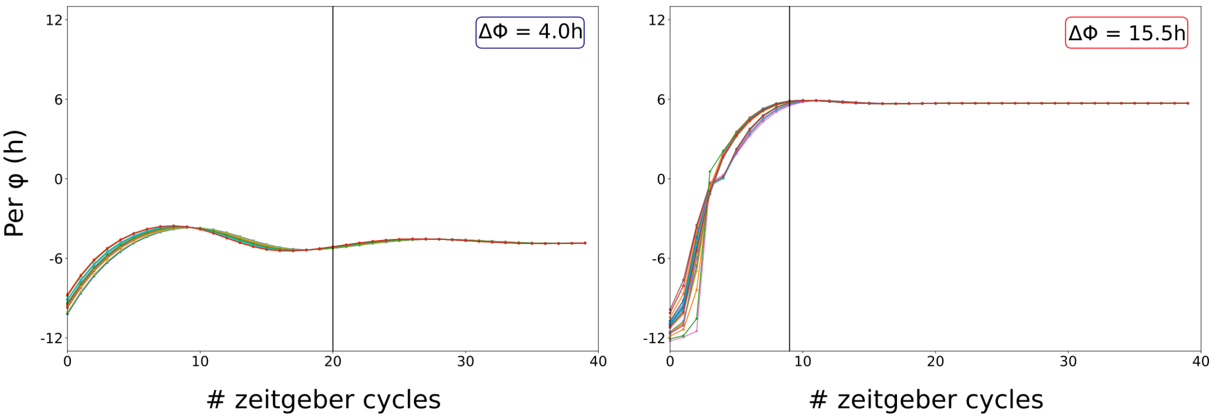

Figure S3-7: Comparison of the transient times to entrainment in the modified Korenčič model for $\Delta\Phi =$ 4.0h (20 zeitgeber cycles, left plot) and $\Delta\Phi = 15.5$h (9 zeitgeber cycles, right plot). In the simulations both zeitgebers have equal periods of 24h and equal zeitgeber amplitudes $r = 0.2$, the median transient times of 24 randomly chosen starting conditions are indicated by vertical lines.

REFERENCES

Granada, A. E. and Herzel, H. (2009). How to achieve fast entrainment? the timescale to synchronization. *PLOS ONE*, 4(9):1–10.
